# Assessing Pulmonary Function in Children and Adolescents After Cancer Treatment: Protocol for a Multicenter Cohort Study (Swiss Childhood Cancer Survivor Study FollowUp–Pulmo)

**DOI:** 10.2196/69743

**Published:** 2025-04-08

**Authors:** Maša Žarković, Christina Schindera, Grit Sommer, Christine Schneider, Jakob Usemann, Maria Otth, Sonja Lüer, Marc Ansari, Philipp Latzin, Claudia E Kuehni

**Affiliations:** 1 Institute of Social and Preventive Medicine University of Bern Bern Switzerland; 2 Graduate School for Health Sciences University of Bern Bern Switzerland; 3 Department of Pediatric Oncology and Hematology University Children’s Hospital Basel University of Basel Basel Switzerland; 4 Graduate School for Cellular and Biomedical Sciences University of Bern Bern Switzerland; 5 Division of Pediatric Hematology and Oncology University Hospital of Bern University of Bern Bern Switzerland; 6 Division of Pulmonology University Children’s Hospital Basel University of Basel Basel Switzerland; 7 Division of Pediatric Respiratory Medicine and Allergology University Hospital of Bern University of Bern Bern Switzerland; 8 Department of Respiratory Medicine University Children's Hospital Zurich University of Zurich Zurich Switzerland; 9 Children’s Research Center University Children's Hospital Zurich University of Zurich Zurich Switzerland; 10 Department of Oncology University Children's Hospital Zurich University of Zurich Zurich Switzerland; 11 Faculty of Health Sciences and Medicine University of Lucerne Lucerne Switzerland; 12 Division of Hematology/Oncology Children's Hospital of Eastern Switzerland St. Gallen Switzerland; 13 Division of Pediatric Oncology and Hematology Department of Women, Child and Adolescent University Hospital of Geneva Geneva Switzerland; 14 CANSEARCH Research Platform for Pediatric Oncology and Hematology Department of Pediatrics, Gynecology and Obstetrics Faculty of Medicine, University of Geneva Geneva Switzerland

**Keywords:** childhood cancer survivors, respiratory function tests, late effects, pulmonary toxicity, multiple breath washout test, cohort study

## Abstract

**Background:**

Childhood cancer survivors (CCS) are at risk of pulmonary dysfunction due to cancer treatments, but evidence on prevalence and risk factors remains limited. Most previous studies had small sample sizes or retrospective study designs, little information about treatments, or a lack of standardization of pulmonary function tests (PFTs) or limited their investigation to certain PFTs. Since spirometry mainly assesses the large airways but cancer therapy also affects peripheral airways, additional functional tests are needed. The nitrogen multiple breath washout test (N_2_MBW) is sensitive to peripheral airway damage in other patient populations, but its benefit in CCS is unknown. Therefore, comprehensive and standardized evaluation of pulmonary function after cancer treatment in childhood, using different PFTs that include N_2_MBW, is needed to address these knowledge gaps and provide insights into possible early stages of pulmonary dysfunction.

**Objective:**

In the Swiss Childhood Cancer Survivor Study (SCCSS) FollowUp–Pulmo, we will comprehensively assess lung function in children and adolescents after treatment for cancer to identify risk factors for pulmonary dysfunction, assess the ability of N_2_MBW to detect pulmonary dysfunction compared to other PFTs, and investigate the association of functional outcomes from PFTs with self-reported respiratory symptoms.

**Methods:**

SCCSS FollowUp–Pulmo is a prospective multicenter longitudinal cohort study embedded in routine clinical care that enrolls CCS aged 6-20 years for whom at least 1 year has passed since a childhood cancer diagnosis, who have completed treatment, and who attend regular pediatric oncological follow-up care. Inclusion criteria comprise any of the following: systemic anticancer treatment (chemotherapy, immunotherapy, or targeted agents), thoracic surgery, thoracic radiotherapy, or hematopoietic stem cell transplantation (HSCT). CCS undergo a standardized pulmonary assessment, including spirometry, body plethysmography, diffusing capacity of the lung for carbon monoxide (DLCO), and N_2_MBW, and complete a questionnaire on respiratory symptoms and lifestyle. Data from previous and subsequent routine care PFTs will be included in this study.

**Results:**

Recruitment started in June 2022 at the University Children’s Hospital Bern, Switzerland. Subsequently, patient recruitment expanded to the University Children’s Hospitals in Basel and Geneva, Switzerland. By October 2024, we had invited 220 patients, of which 201 have already participated in this study, resulting in a response rate of 91%. Their median age at the time of the study was 14 years (IQR 10-17), and the median time since diagnosis was 7 years (IQR 4-10). The study will continuously enroll new CCS.

**Conclusions:**

This study will contribute to a comprehensive understanding of pulmonary function in CCS and assess related risk factors, as well as the utility of N_2_MBW compared to other PFTs. The results will assist in the development of more targeted screening and risk-stratified follow-up care.

**Trial Registration:**

ClinicalTrials.gov NCT04732273; https://clinicaltrials.gov/study/NCT04732273

**International Registered Report Identifier (IRRID):**

DERR1-10.2196/69743

## Introduction

### Survival Rates and Long-Term Complications

Advances in childhood cancer treatment and supportive care have resulted in survival rates that now exceed 80% in high-income countries [1]. Yet, even as cancer treatments are curative in targeting cancer cells, they can harm healthy tissue and potentially cause late complications, such as second neoplasms and chronic diseases [2,3]. The cumulative incidence of such late effects among childhood cancer survivors (CCS) thus predisposes them to increased morbidity and premature mortality [4]. Among late effects, pulmonary complications are the third-leading cause of excess mortality after second neoplasms and cardiovascular diseases [5].

### Pulmotoxic Treatments

Several cancer treatment modalities can be pulmotoxic. These include the chemotherapeutic agents busulfan, bleomycin, carmustine, and lomustine; thoracic radiotherapy; thoracic surgery; and hematopoietic stem cell transplantation (HSCT) [6,7]. The underlying mechanisms of pulmonary toxicity involve alveolar, vascular, and parenchymal damage resulting from chemotherapy and radiotherapy, which may progress to lung fibrosis [6-8]. HSCT-related lung damage can result from intensive conditioning regimens, infections, or graft-versus-host disease (GvHD), while surgery of the chest or lungs may reduce lung volumes and impair chest wall compliance [7,9]. However, recently published recommendations for surveillance of pulmonary function among CCS from the International Late Effects of Childhood Cancer Guideline Harmonization Group (IGHG) could not consistently confirm the pulmonary toxicity of all these treatments, particularly certain chemotherapeutics, due to limited and low-quality evidence [10]. Other chemotherapeutics, such as methotrexate and cyclophosphamide, have also been suspected of causing lung damage [8,11], but findings across studies remain inconsistent [12,13]. Considering that these treatments may harm developing lungs and potentially lead to progressive pulmonary damage [14], it is important to study their effects on lung function.

### Prevalence and Detection of Pulmonary Dysfunction

Pulmonary dysfunction in CCS exposed to pulmotoxic treatments has been reported in varying proportions of CCS (44%-77%), depending on study populations and criteria used to define obstructive, restrictive, and diffusion impairments [12,13,15,16]. The lung has a large functional reserve, and early disease may often remain asymptomatic, particularly when it affects the lung periphery [17]. Most studies have used conventional pulmonary function tests (PFTs), such as spirometry, body plethysmography, and diffusing capacity of the lung for carbon monoxide (DLCO), to assess lung function in CCS. However, spirometry and body plethysmography lack the sensitivity to detect changes in small airways [18], which may be damaged first [6]. The nitrogen multiple breath washout test (N_2_MBW), which measures ventilation inhomogeneity of the ventilated lung, detecting small airway disease, has been increasingly used in other patient populations, including those treated with allogeneic HSCT [19-21]. In a small prospective study of adult CCS, N_2_MBW identified more cases of pulmonary dysfunction than spirometry, even among those who had not been exposed to previously defined pulmotoxic treatments [22]. Though larger prospective studies with standardized assessments are still needed, these findings suggest that N_2_MBW could be a valuable complementary test in screening CCS for early pulmonary damage.

### Current Knowledge Gaps

The recently published IGHG recommendations not only summarized existing evidence but also highlighted large knowledge gaps and methodological weaknesses in previous research [10]. These gaps emphasize the need for prospective, longitudinal studies with larger sample sizes and a broader range of treatment exposures to characterize the onset and progression of treatment-related pulmonary dysfunction. The long-term effects of newer chemotherapeutic and immunotherapeutic agents on lung function are understudied [23]. Evidence on how treatment-related complications, comorbidities, and genetic variants influence lung damage risk is limited as well. Similarly, standardization is lacking in PFTs and in the use of appropriate age- and sex-specific reference values. For example, results should be reported as z-scores rather than just proportions of patients with reduced lung function. Additionally, few studies have assessed diagnostic tests specific to the location and type of potential dysfunction, such as N_2_MBW for peripheral inhomogeneity in ventilation. More data are also needed on the association between PFT outcomes and clinical symptoms. To address these gaps, we designed the Swiss Childhood Cancer Survivor Study (SCCSS) FollowUp–Pulmo.

### Study Objectives

The primary objective of SCCSS FollowUp–Pulmo is to longitudinally investigate lung function in children and adolescents after cancer treatment using a comprehensive set of PFTs that also assess small-airway disease. Second, the study will investigate possible effects of treatment-related risk factors (systemic anticancer agents, thoracic radiotherapy, thoracic surgery, and HSCT), treatment-related complications (pulmonary infections, GvHD), and existing comorbidities (eg, pulmonary or cardiac disease) on lung function. Third, the study will examine the ability of N_2_MBW to detect pulmonary dysfunction in comparison with other PFTs. Fourth, it will investigate the association between lung function and self-reported respiratory symptoms.

## Methods

### Study Design and Inclusion Criteria

SCCSS FollowUp–Pulmo is a multicenter prospective longitudinal cohort study integrated into the routine clinical care of several children’s hospitals in Switzerland. The study is an interdisciplinary collaboration between the pediatric hematology/oncology and pediatric pulmonology departments of respective centers, the Institute of Social and Preventive Medicine (ISPM) at the University of Bern, and the Swiss Childhood Cancer Registry (ChCR). The ChCR is a nationwide, population-based cancer registry that includes Swiss residents diagnosed up to the age of 20 years with leukemia, lymphoma, central nervous system (CNS) tumors, malignant solid tumors, or Langerhans cell histiocytosis, classified according to the *International Classification of Childhood Cancer, Third Edition* (ICCC-3) [24]. Although the ChCR captures over 95% of children diagnosed in Switzerland [25], SCCSS FollowUp–Pulmo will also include a few patients treated and followed up in Swiss clinics who may not be registered in the ChCR due either to registration delays or residency outside of Switzerland.

All CCS aged 6-20 years for whom 1 year or more has elapsed since cancer diagnosis, who have completed treatment, and who are in regular pediatric hemato-oncological follow-up care are eligible for SCCSS FollowUp–Pulmo. The following treatment modalities possibly affecting lung function are included in SCCSS FollowUp–Pulmo: any systemic anticancer treatment (chemotherapy, immunotherapy, or targeted therapy) [26]; thoracic surgery involving the chest or lungs (excluding central line placement) [27]; radiation of the lungs, the chest (axilla, mantle, mediastinal), or scattered radiation from other radiation fields, including the whole abdomen or any upper abdominal field, spinal doses of 30 Gray or higher, and total body irradiation [27]; and HSCT [28]. Excluded are CCS who were treated only with surgery or radiation outside the thorax due to their low risk for pulmonary dysfunction [29] and patients with relapse or in palliative care at the time of recruitment.

### Ethical Considerations

Ethical approval was granted by the Ethics Committee of the Canton of Bern, Switzerland (KEK-BE: 2019-00739), and the study is registered on ClinicalTrials.gov (NCT04732273). All participants provide a signed informed consent form. If any patient declines to participate, their data are not collected. Data are entered into the Research Electronic Data Capture (REDCap) database version 14.0.10 (Vanderbilt University), which complies with legal requirements for data security and data protection.

### Study Procedures

The study is coordinated by the ISPM research team, which is responsible for overall study management, monitoring recruitment progress, and handling administrative and financial aspects. Clinical teams at each participating center include pediatric oncologists, pulmonologists, data managers, and study nurses.

#### Step 1: Selection of Eligible CCS

**Figure 1:**
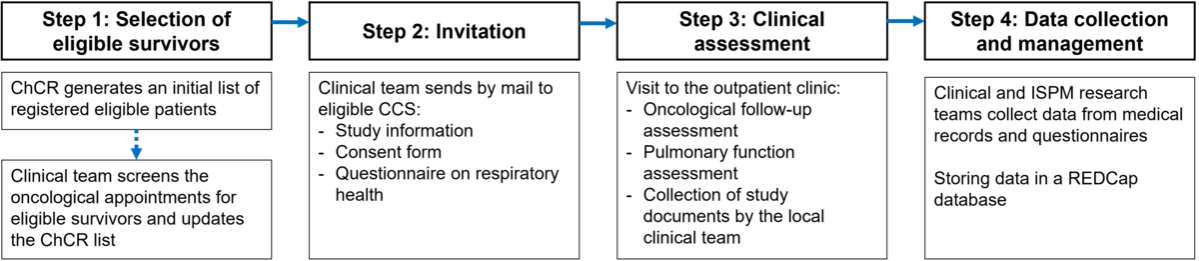
Flowchart of SCCSS FollowUp–Pulmo procedures. CCS: childhood cancer survivors; ChCR: Swiss Childhood Cancer Registry; ISPM: Institute of Social and Preventive Medicine; REDCap: Research Electronic Data Capture.

Eligible CCS are identified in 2 ways: (1) the ChCR provides an initial list of eligible patients to participating hospitals, and (2) a member of the clinical team regularly screens upcoming follow-up appointments, cross-referencing with the ChCR and identifying any additional eligible patients (Figure 1). This process ensures the inclusion of all eligible patients. The treating physician organizes pulmonary function assessments for the upcoming oncological follow-up appointment. Detailed recruitment procedures are developed at each center to consider clinical workflows.

#### Step 2: Invitation

The clinical team sends the study information, consent form, and a questionnaire on respiratory health to eligible survivors prior to their next oncological follow-up appointment that will include PFTs. CCS who consent to participate send the documents back or bring them to the consultation.

#### Step 3: Clinical Assessment

**Figure 2:**
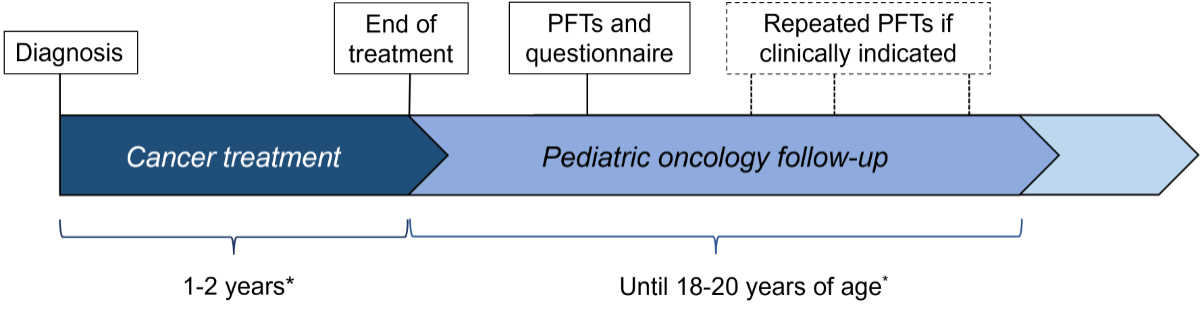
Timeline of pulmonary function assessments during SCCSS FollowUp–Pulmo. *The duration of cancer treatment varies based on the specific cancer diagnosis and the corresponding treatment protocol, while the age at which a patient transitions out of pediatric oncology follow-up may vary individually, depending on the cancer treatment protocol and the practices of the respective center. PFT: pulmonary function test; SCCSS: Swiss Childhood Cancer Survivor Study.

At the oncological follow-up assessment, patients first meet their pediatric oncologist, who obtains a history, performs a physical examination, and refers them to pediatric pulmonology for PFTs. Since the PFTs are scheduled within the follow-up care, they are conducted irrespective of study consent. Patients with pathological results will undergo repeated PFTs, as clinically indicated (Figure 2). The clinical team collects the signed consent form and questionnaire. If patients do not complete the consent form and questionnaire prior to or during the clinical visit, they can still return the documents later. If a patient declines to participate, no data are collected for study purposes.

#### Step 4: Data Collection and Management

The clinical team at the respective study centers and the research team at the ISPM extract the data of each consenting participant from medical records, PFTs, and questionnaires. All data are stored in the REDCap database. In the database, each patient has a unique REDCap ID.

### Exposures and Outcomes of Interest

#### Pulmonary Function Tests

CCS undergo a set of PFTs, including spirometry, body plethysmography, DLCO, and N_2_MBW, according to the American Thoracic Society/European Respiratory Society (ATS/ERS) recommendations [30,31]. PFTs are conducted in pediatric lung function laboratories by trained technicians. Standard operating procedures for PFTs are harmonized among centers to ensure comparability, with quality control performed by lung function technicians. Because N_2_MBW is not yet widely used in clinical settings, centralized quality control is additionally performed to ensure that only high-quality tests and the latest algorithms are included. Specialized software developed by experts at the University Hospital in Bern detects evidence of leaks, insufficient waiting time between tests, early termination of tests, synchronization issues, and abnormal breathing patterns or volumes in accordance with consensus guidelines [32,33]. Experienced pulmonologists interpret and review the results of PFTs.

Since asthma and allergic conditions are common differential diagnoses in young patients, including CCS, most clinics also measure fractional exhaled nitric oxide (FeNO) in those with obstructive patterns on spirometry or asthma-like symptoms [34]. High FeNO levels suggest an asthmatic or allergic etiology, while low levels may indicate noninflammatory causes related to cancer treatment effects.

Table 1 shows the main outcome measures from PFTs, their interpretation, anatomical correlates, and studies for reference values used for respective PFTs. SCCSS FollowUp–Pulmo also collects all prestudy PFT data from patients who previously underwent these assessments and subsequent PFTs conducted as part of ongoing clinical care.

**Table 1:** PFTs^a^ performed in CCS^b^, their interpretation, and anatomical correlates.

Test	Main outcomes	Meaning of abnormal results	Anatomical correlates	Reference values
Spirometry	FVC^c^, FEV_1_^d^, FEV_1_/FVC, FEF_25%-75%_^e^	Airway obstruction, reduced dynamic lung volume	Fibrotic destruction of lung tissue and large airways, reduced lung compliance	Quanjer et al [35]
Body plethysmography	FRC^f^, SR_eff_^g^, SR_tot_^h^, RV^i^, TLC^j^, VC^k^	Reduced static lung volume, hyperinflation	Fibrotic destruction of lung tissue and large airways, reduced lung compliance	Hall et al [36]
DLCO^l^	DLCO	Reduced alveolar-capillary gas transfer, reflected by diffusion deficits	Alveolar-capillary membrane damage	Stanojevic et al [37,38]
N_2_MBW^m^	LCI^n^, S_ACIN_^o^, S_COND_^p^	Increased ventilation inhomogeneity of airways with reduced global, alveolar, and conducting ventilation	Fibrotic damage of small airways	Ramsey et al [39]
FeNO^q^	FeNO	Eosinophilic airway inflammation as a key component of allergic asthma	Allergic inflammation as an alternative cause of pulmonary obstruction	Jacinto et al [34]

^a^PFT: pulmonary function test.

^b^CCS: childhood cancer survivors.

^c^FVC, forced vital capacity.

^d^FEV_1_, forced expiratory volume in 1 second.

^e^FEF_25%-75%,_ forced expiratory flow at 25%-75% of the FVC.

^f^FRC, functional residual capacity.

^g^SR_eff_: specific effective resistance.

^h^SR_tot_: specific total resistance.

^i^RV: residual volume.

^j^TLC: total lung capacity.

^k^VC: vital capacity.

^l^DLCO: diffusion capacity of the lung for carbon monoxide.

^m^N_2_MBW: nitrogen multiple breath washout test.

^n^LCI: lung clearance index.

^o^S_COND_: conductive ventilation inhomogeneity index.

^p^S_ACIN_: acinar ventilation inhomogeneity index.

^q^FeNO: fractional exhaled nitric oxide (measured in CCS with symptoms suggestive of asthma, eg, wheeze, dyspnea, cough, or signs of obstruction in spirometry).

#### Medical and Questionnaire Data

Information obtained from medical records includes anthropometric measures, respiratory disease history, physical evaluation, PFTs, cancer diagnosis and treatment, and additional data from the medical history, including comorbidities and significant treatment-related complications (GvHD, pulmonary infections) (Table 2). CCS complete a detailed questionnaire on respiratory health that includes sections on respiratory symptoms, infectious diseases, exercise-induced problems, allergic and pulmonary diseases, family history of respiratory conditions, lifestyle and environmental factors, and sociodemographic information.

**Table 2:** Description of medical and questionnaire data collected as part of SCCSS^a^ FollowUp–Pulmo.

Data source and data items		Description
**Medical records**		
	Personal information and anthropometric measures	Date of birth Sex Height Weight BMI
	Respiratory history and physical evaluation	Recent history of airway infections Lung auscultation Thoracic inspection Signs of dyspnea Oxygen saturation
	PFTs^b,c^	Spirometry Body plethysmography DLCO^d^ N_2_MBW^e^ FeNO^f^
	Cancer diagnosis	Date of diagnosis Type of cancer and location Metastases Relapse Second malignant neoplasm
	Cancer treatment	Treatment protocol and arm Start and end dates Cumulative doses of all individual chemotherapy drugs, targeted agents, and immunotherapies Radiotherapy (cumulative dose, location, duration) Surgery (location, type) HSCT^g^ (autologous or allogeneic, donor type and source, conditioning regimens, complications)
	Additional data from medical history	GvHD^h^ (acute or chronic, affected organs, grade, treatment) Significant pulmonary infections during or after cancer treatment (diagnosis, causing pathogen, duration of hospitalization) Comorbidities
**Questionnaire**		
	Respiratory symptoms	Cough (type, duration, with or without a cold) Wheeze (frequency, duration, triggers) Dyspnea(frequency, duration, triggers)
	Infectious diseases^i^	Otitis Sinusitis Pneumonia
	Exercise-induced problems	Frequency Types Triggering situations
	Allergic diseases	Allergic rhinitis Hay fever Atopic dermatitis
	Pulmonary diseases^j^	Asthma Bronchitis Lung fibrosis Emphysema
	Lifestyle and environment	Physical activity (compulsory school sport, recreational sport) Active and passive smoking (amount and type of tobacco products)
	Sociodemographic data and family history	Citizenship Parental education and profession Family history of asthma, chronic bronchitis, hay fever, and atopic dermatitis

^a^SCCSS: Swiss Childhood Cancer Survivor Study.

^b^PFT: pulmonary function test.

^c^For each pulmonary function test, the date, test quality, and multiple outcomes (as listed in Table 1) are recorded.

^d^DLCO: diffusing capacity of the lungs for carbon monoxide.

^e^N_2_MBW: nitrogen multiple breath washout test.

^f^FeNO: fractional exhaled nitric oxide.

^g^HSCT: hematopoietic stem cell transplantation.

^h^GvHD: graft-versus-host disease.

^i^Data includes the recurrence and treatment of each disease.

^j^Data includes the treatment of each disease.

### Sample Size Calculation

To determine the sample size needed for our study, we based calculations on the study by Schindera et al [22], who assessed lung function in long-term CCS using spirometry and N_2_MBW. This single-center study was conducted on Swiss CCS and applied the same inclusion criteria for treatment exposures, making it a suitable reference. The main outcome from N_2_MBW was the lung clearance index (LCI). The mean LCI z-score was 1.37 (SD 2.69). We calculated the number of participants necessary to achieve a statistical significance level of 0.05 and a power of 0.80, while accounting for a 15% dropout rate. This calculation indicated that a minimum of 146 participants would be required to detect a similar deviation in LCI with sufficient statistical power. We plan to include a larger sample size to increase precision, improve our ability to detect smaller deviations, and ensure adequate power to analyze other lung function outcomes and investigate specific subgroups of patients defined by tumor type and treatments received.

### Statistical Analysis

To compare PFT outcomes with normal values, we will calculate z-scores and percent-predicted values using external Global Lung Initiative (GLI) reference data [35-37,39]. We will define pulmonary dysfunction as z-scores below –1.64 or above +1.64 for respective PFT indices since these thresholds represent deviations from the reference population mean and indicate abnormalities in pulmonary function. To characterize pulmonary function among CCS, we will analyze z-scores for predefined outcomes and assess differences based on characteristics such as treatment exposure, age at treatment, and time since cancer diagnosis. We will assess group differences using appropriate statistical tests based on the type of variable, such as the *t*-test or the Mann-Whitney test for continuous variables and the *χ*² test or the Fisher exact test for categorical variables. For analyses of associations between outcomes and covariates, we will apply regression models adjusted for potential confounders. For longitudinal data, we will use mixed effects models to account for repeated measures over time. We will use Stata (Stata Corp LLC) and R (Foundation for Statistical Computing) for statistical analyses.

## Results

**Figure 3:**
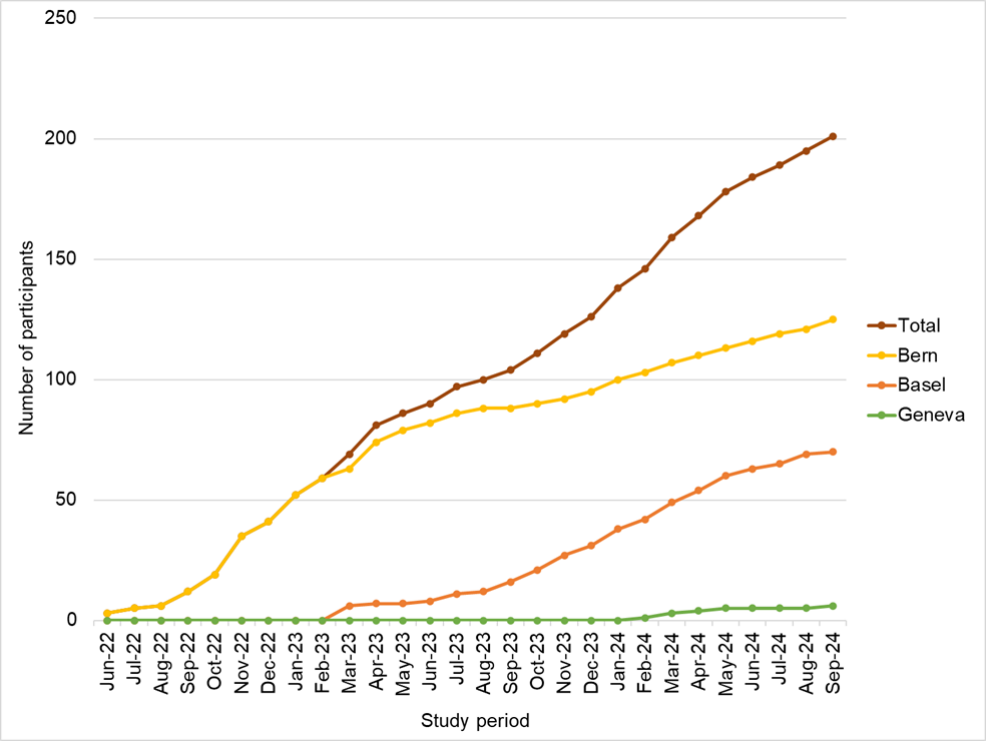
Number of participants in SCCSS FollowUp–Pulmo since the start of the study. SCCSS: Swiss Childhood Cancer Survivor Study.

Recruitment started in June 2022 at the University Children’s Hospital in Bern, in March 2023 at the University Children’s Hospital in Basel, and in March 2024 at the University Children’s Hospital in Geneva. The number of new participants per month varies across the centers, depends on clinical capacity, and is steadily growing (Figure 3). As of October 2024, a total of 220 patients had been invited to participate in the study. Of those, 201 patients consented to and underwent PFTs, resulting in a response rate of 91%. Bern registered 125 (62%) participants, Basel 70 (35%), and Geneva 6 (3%). The time required to perform all PFTs and complete the questionnaire was 45-60 minutes per participant.

More than half of the 201 participants (n=119, 59%) were male, the median age at the time of the study was 14 years (IQR 10-17), and the median time since diagnosis was 7 years (IQR 4-10), as shown in Tables 3 and 4. The most common diagnoses were leukemia (n=105, 52%), lymphoma (n=22, 11%), and neuroblastoma (n=18, 9%). All but 2 (1%) participants had been treated with chemotherapy, 25 (13%) had received thoracic radiotherapy, and 15 (8%) had undergone thoracic surgery. In total, 20 (10%) participants had undergone HSCT, with 11 (6%) having been treated with autologous and 9 (4%) with allogeneic HSCT.

**Table 3:** Demographic characteristics of CCS^a^ participating in SCCSS^b^ FollowUp–Pulmo up to October 2024.

Characteristics		Participants (N=201)
**Sex, n (%)**		
	Male	119 (59)
	Female	82 (41)
Age at the time of study (years), median (IQR)		14 (10-17)
**Age group (years), n (%)**		
	6-10	47 (23)
	11-14	73 (36)
	15-18	66 (33)
	≥19	15 (8)

^a^CCS: childhood cancer survivors.

^b^SCCSS: Swiss Childhood Cancer Survivor Study.

**Table 4:** Clinical characteristics of CCS^a^ participating in SCCSS^b^ FollowUp–Pulmo up to October 2024.

Characteristics		Participants (N=201)
Age at diagnosis (years), median (IQR); range		5 (3-9); 0.1-17
Time since diagnosis (years), median (IQR); range		7 (4-10); 1-17
**Time since diagnosis (years), n (%)**		
	<5	68 (34)
	5-10	83 (41)
	11-15	41 (20)
	>15	9 (5)
**Diagnosis (ICCC-3^c^), n (%)**		
	Leukemia	105 (52)
	Lymphoma	22 (11)
	CNS^d^ tumor	11 (6)
	Neuroblastoma	18 (9)
	Retinoblastoma	0
	Renal tumor	15 (8)
	Hepatic tumor	2 (1)
	Bone tumor	6 (3)
	Soft tissue sarcoma	14 (7)
	Germ cell tumor	2 (1)
	Other tumor^e^	2 (1)
	Langerhans cell histiocytosis	4 (2)
**Chemotherapy^f^ (n=199, 99%), n (%)**		
	Pulmotoxic chemotherapy	16 (8)
**Other treatments, n (%)**		
	Thoracic radiotherapy^g^	25 (13)
	Thoracic surgery^h^	15 (8)
**HSCT^i^ (n=20, 10%), n (%)**		
	Autologous	11 (6)
	Allogeneic	9 (5)
Relapse, n (%)		15 (8)

^a^CCS: childhood cancer survivors.

^b^SCCSS: Swiss Childhood Cancer Survivor Study.

^c^*ICCC-3*: *International Classification of Childhood Cancer, Third Edition*.

^d^CNS: central nervous system.

^e^Other malignant epithelial neoplasms, malignant melanomas, and other/unspeciﬁed malignant neoplasms.

^f^Any chemotherapy alone or combined with other treatments.

^g^Radiotherapy involving chest, abdomen, spine, or total body irradiation alone or combined with other treatments.

^h^Surgery involving the thorax or lungs alone or combined with other treatments.

^i^HSCT: hematopoietic stem cell transplantation alone or combined with other treatments.

## Discussion

### Summary

This prospective, multicenter cohort study of lung function in children and adolescents after cancer treatment investigates risk factors, compares the detection of pulmonary dysfunction using N_2_MBW and conventional PFTs, and examines the association of functional outcomes from PFTs with respiratory symptoms.

### Comparison With Previous Research

Previous studies of pulmonary dysfunction after treatment for childhood cancer have mostly been retrospective, based on chart reviews [14,40-42]. This has entailed methodological weaknesses, particularly a risk of selection bias because CCS for whom PFT results were obtained might overrepresent symptomatic CCS or those treated more intensively. We identified several prospective studies on pediatric CCS, but like the retrospective ones, they mostly recruited specific groups of patients treated with previously defined pulmotoxic treatments, such as HSCT [42-44] or thoracic radiation [14,45], or specific tumor types like leukemia [46] or lymphoma [47,48]. Data on the pulmotoxic effects of individual agents, such as busulfan [49], melphalan [50], cyclophosphamide [46,50], and methotrexate [51], remain limited. By including CCS exposed to systemic therapies, including any chemotherapy, targeted agents, or immunotherapy, our study will provide a better understanding of treatment-related pulmonary dysfunction in a broadly representative population of CCS. Continuous recruitment will allow us to collect data on the pulmonary effects of newer treatments used in contemporary protocols, whose impacts remain largely unknown [52]. Detailed information from medical records will allow the investigation of effects of comorbidities and treatment-related complications on PFT outcomes.

Previous studies have rarely included sensitive tests, such as N_2_MBW, that can detect early changes in the lung periphery. Most have used spirometry, body plethysmography, and DLCO [12,14,15,49]. A study investigating 57 pediatric CCS with a median follow-up time of 6.2 years from end of treatment did not find differences in ventilation inhomogeneity measured using N_2_MBW compared to healthy controls [53]. In contrast, several studies on patients after allogeneic HSCT have reported the LCI to be a sensitive measure for early pulmonary complications [20,21,54]. This study will obtain N_2_MBW data for CCS exposed to a wide range of treatment modalities and investigate whether N_2_MBW is more sensitive than other PFTs in detecting early pulmonary dysfunction.

Another drawback of many existing studies is that they report PFT data using binary cut-offs and describe results as either normal or abnormal [12,15,49]. This reduces statistical power and introduces interpretations based on predefined threshold values. Reporting PFT results as raw data and z-scores based on internationally agreed-upon, age-adjusted reference values will allow better comparison and pooling across studies. Limited data exist on how lung function correlates with clinical symptoms [10]. Questionnaire data collected in this study will help investigate symptoms and other patient-reported outcomes, as well as their correlation with PFT results.

### Collaboration With Other Ongoing Studies

The SWISS-Pearl Study (ClinicalTrials.gov ID: NCT05427136), currently conducted in multiple centers in Switzerland, investigates lung function in patients with pediatric cancer. The study includes spirometry, body plethysmography, N_2_MBW, magnetic resonance imaging of the lungs, and questionnaires at different points during cancer treatment. We plan to combine that study with ours to create a comprehensive database, enabling us to analyze lung function trajectories in patients and survivors of pediatric cancer.

The GECCOS (Genetic Risks for Childhood Cancer Complications Switzerland) study is a nationwide cohort study collecting germline genetic data from patients and survivors of childhood cancer in Switzerland [55]. In consenting patients, we will link clinical and PFT data with the genetic data from GECCOS, allowing us to investigate the effects of genetic predisposition on pulmonary toxicity. This will assist the development of personalized treatment strategies and risk-adapted long-term care for survivors.

### Study Limitations

A current limitation of this study is the limited number of participants and the heterogeneity of the study population, which can limit the statistical power for conducting subgroup analyses. For instance, exploring rare tumors or assessing specific effects of individual chemotherapeutic agents may at present be challenging. However, we plan to expand the study to more centers and pool data with international collaborations. Another limitation is the lack of systematic baseline PFT assessments before cancer treatment, making it difficult to distinguish treatment-related pulmonary dysfunction from preexisting conditions. Future studies should include pretreatment PFTs to better track changes in z-scores over time and improve the assessment of therapy-related effects. Finally, there is a small risk of selection bias because CCS with a longer time since the end of treatment, who do not experience respiratory symptoms or were not exposed to pulmotoxic treatments, may be less likely to participate in the study, potentially leading to underrepresentation of healthy CCS. Yet, because the study is embedded in regular follow-up care and supported by oncologists, with initial results showing a high participation rate, exceeding 90%, this bias should be minimal.

### Conclusion

This multicenter cohort study prospectively investigates pulmonary dysfunction in young CCS. By assessing lung function as an intermediate outcome, rather than established disease or mortality, this study will provide a resource for evaluating pulmonary dysfunction at an earlier stage in the disease trajectory, particularly within the early years posttreatment. The initial response shows that integrating standardized pulmonary evaluations into routine follow-up care in Switzerland is feasible and widely accepted by both survivors and health care providers. The findings of this study will provide new insights to inform the development of guidelines and recommendations for pulmonary follow-up care.

## Abbreviations

CCS: childhood cancer survivors
ChCR: Swiss Childhood Cancer Registry
CNS: central nervous system
DLCO: dffusing capacity of the lung for carbon monoxide
FeNO: fractional exhaled nitric oxide
GECCOS: Genetic Risks for Childhood Cancer Complications Switzerland
GvHD: graft-versus-host disease
HSCT: hematopoietic stem cell transplantation
ICCC-3: International Classification of Childhood Cancer, Third Edition
IGHG: International Late Effects of Childhood Cancer Guideline Harmonization Group
ISPM: Institute of Social and Preventive Medicine
LCI: lung clearance index
N_2_MBW: nitrogen multiple breath washout test
PFT: pulmonary function test
REDCap: Research Electronic Data Capture
SCCSS: Swiss Childhood Cancer Survivor Study

## Data Availability

Researchers interested in collaborative work can contact the corresponding author (CK) to discuss planned projects.
